# AphanoDB: a genomic resource for *Aphanomyces *pathogens

**DOI:** 10.1186/1471-2164-8-471

**Published:** 2007-12-20

**Authors:** Mohammed-Amine Madoui, Elodie Gaulin, Catherine Mathé, Hélène San Clemente, Arnaud Couloux, Patrick Wincker, Bernard Dumas

**Affiliations:** 1 UMR 5546 CNRS Université Paul Sabatier Toulouse III Pôle de Biotechnologie Végétale 24, Chemin de Borde-Rouge BP 42617, Auzeville 31326 Castanet-Tolosan, France; 2 UMR 8030 Génoscope – CNRS, 2 rue Gaston Crémieux, 91000 Evry, France

## Abstract

**Background:**

The Oomycete genus *Aphanomyces *comprises devastating plant and animal pathogens. However, little is known about the molecular mechanisms underlying pathogenicity of *Aphanomyces *species. In this study, we report on the development of a public database called AphanoDB which is dedicated to *Aphanomyces *genomic data. As a first step, a large collection of Expressed Sequence Tags was obtained from the legume pathogen *A. euteiches*, which was then processed and collected into AphanoDB.

**Description:**

Two cDNA libraries of *A. euteiches *were created: one from mycelium growing on synthetic medium and one from mycelium grown in contact to root tissues of the model legume *Medicago truncatula*. From these libraries, 18,684 expressed sequence tags were obtained and assembled into 7,977 unigenes which were compared to public databases for annotation. Queries on AphanoDB allow the users to retrieve information for each unigene including similarity to known protein sequences, protein domains and Gene Ontology classification. Statistical analysis of EST frequency from the two different growth conditions was also added to the database.

**Conclusion:**

AphanoDB is a public database with a user-friendly web interface. The sequence report pages are the main web interface which provides all annotation details for each unigene. These interactive sequence report pages are easily available through text, BLAST, Gene Ontology and expression profile search utilities. AphanoDB is available from URL: .

## Background

Oomycetes form a phylogenetically distinct group of eukaryotic microorganisms which includes plant and animal pathogens, that cause widespread damages of high economical [1-3] and ecological impacts [4]. Pathogenic oomycete species are found mainly in three orders, the Pythiales, the Peronosporales and the Saprolegniales [5]. From recent studies on the phylogenic relationships within oomycetes, it has been suggested that the ability to infect plants appeared at least twice in the oomycete lineage, first in an ancient lineage which evolved into the Pythiales (including *Phytophthora *and *Pythium*) and Peronosporales, and secondly in the Saprolegniales lineage [6], which includes destructive animal pathogens such as the fish pathogens *Saprolegnia parasitica *and *Aphanomyces piscida*, and plant pathogens such as *A. euteiches *and *A. cochlioides*. Among members of Oomycetes, *Phytophthora *is the best studied genus and genomic resources are available for several species (cDNA libraries and/or complete genome sequence) [7-11]. In contrast, few nucleic acid sequences are available from species classified in the Saprolegniales, the main data consisting in a 1510 ESTs collection obtained from the fish pathogen *S. parasitica *[12].

Recently, we have developed a pathosystem which consists of the model legume *Medicago truncatula *and the legume pathogen *A. euteiches *[13]. Up to now, no chemical compound is able to protect efficiently against *A. euteiches *and infested fields cannot be used any longer for legume production during many years [14]. In order to get a better knowledge of the *A. euteiches*-legume interaction and to identify molecular targets for drug design, a genomic approache was used. Two unidirectional cDNA libraries from a *A. euteiches *strain ATCC201684 [15] were prepared: one from mycelium growing in a liquid medium containing yeast extract and glucose (MYC library), and one from mycelium in contact to *M. truncatula *root tissues (INT library). The latter situation is a simplified model for growth during pathogenesis. A total of 18,684 expressed sequence tags (9,224 from MYC library and 9,460 from INT library) were submitted to the EBI databank for accession number assignation and were assembled into 7,977 unigenes. Here we present a database named AphanoDB in which all the data were structured. Users can retrieve information using text searches or BLAST analyses. AphanoDB is currently the most extensive resource of *Aphanomyces *sequences and related annotations.

## Construction and content

### Preparation of cDNA libraries and sequencing

The MYC library (saprophytic library) was made with total RNA isolated from mycelium after 5, 7 and 9 days of growth in liquid YG medium (2.5% Yeast extract, 5% Glucose, w/v) at 23°C in the dark. Mycelia were frozen in liquid nitrogen and total RNA was extracted. RNAs of each sample were mixed in equal amounts. mRNAs were purified using an Oligotex mRNA purification kit (Qiagen, Valencia, CA, USA) according to the manufacturer's instructions. A unidirectionnal library was constructed in pSport1 plasmid using the Superscript Plasmid System for cDNA Synthesis and Cloning (Invitrogen, USA). Plasmid ligations were transferred by electroporation into *E. coli *DH5α cells following the manufacturer's protocol (Invitrogen Life Technologies, CA, USA). A library of approximately 5 × 10^5 ^colony forming units was obtained.

The INT library (interaction library) was made with total mRNAs purified from mycelium grown for 1 or 2 days on *M. truncatula *roots. Roots of two week-old plants were laid onto a 2 day-old mycelium, and culture continued for 1 and 2 days. Before the mycelium harvesting, plants were gently removed from the Petri dish to avoid any contamination of *A. euteiches *RNA with plant RNA.*A. euteiches *mRNA extracts were pooled and cDNA generated as described for the MYC library. A library of approximately 5 × 10^5 ^colony forming units was obtained. About 10,000 clones from each library were 5' end sequenced using T7 primer on ABI3730xl DNA Sequencers.

### EST quality and assembling

**Figure 1:**
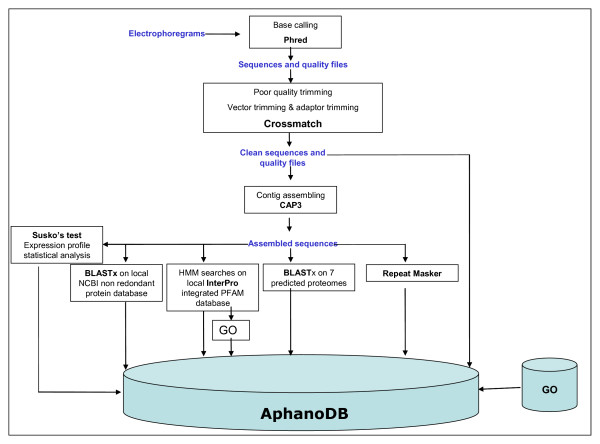
AphanoDB pipeline flow chart.

The two libraries were processed together. All reads were obtained using the Phred program [16]. Only reads with a Phred value over 20 on 80% of the sequences and with a length of over 100 bp were selected. MultiFASTA sequence and quality files were generated and cleaned by vector and adaptor trimming using crossmatch (Figure 1). After these steps 18,684 high-quality sequences were obtained corresponding to 93% of the initial sequence set. These sequences were assembled using the CAP3 program [17]. Minimum overlap length was set to 100 bp, minimum identity percent to 97% and maximum gap length to 30 bp (-o 100 -p 97 -f 30). ESTs from the two libraries were assembled to obtain 7,977 unigenes composed of 2,843 contigs and 5,134 singletons (Table 1). Consensus sequences were renamed with a unique identifier with the prefix 'Ae' for the species, 3 digits for the number of ESTs included in the contig, the 2 letters 'AL' for the strain and 5 digits as individual number.

**Table 1:** Statistics on AphanoDB ESTs status.

**Total number of quality ESTs**	
Library MYC	9,224
Library INT	9,460

**Total number of unigenes**	

Contigs	2,843
Singletons	5,134
Mean number of ESTs per contig	4.73

**Unigenes annotation (%)**	

NCBI nr (E value ≤ 1e-10)	61.5
PFAM (E value ≤ 1e-5)	45
GO db (E value ≤ 1e-5)	41.5

### EST analysis and functional annotation

Assignment of putative functions was performed by running the BLASTX algorithm (release 2.2.14) [18], against a local NCBI non-redundant (nr) protein database (5-17-2007 Version). 61% of the sequences showed similarity to a protein sequence with an E-value ≤ 1e-10. To estimate the level of contamination of *A. euteiches *ESTs with *M. truncatula *cDNA sequences which might occur in the INT library, the unigene sequences were compared to 270,000 *M. truncatula *ESTs deposited in the GenBank database using the BLASTN algorithm. Only 11 unigenes showed a high similarity (E value < 1e-100) to *M. truncatula *ESTs and only two unigenes were composed of ESTs from the INT library. However, these two sequences displayed a higher similarity to *Phytophthora *sequences than to *M. truncatula *sequences. From this analysis, it can be concluded that contamination of the INT library with *M. truncatula *cDNAs is very low if any. Sequences were compared to proteome data from seven different fully sequenced organisms using BLASTX algorithm, since parts of the proteome of these organisms are not present in the nr database. To facilitate comparative analyses between oomycete species, *A. euteiches *sequences were compared to the *P. sojae *and *P. ramorum *proteomes [8]. Since it has been shown that oomycetes are phylogenetically related to diatoms, *A. euteiches *sequences were compared to the *Thalassiosira pseudonana *proteome [19]. In order to find genes putatively involved in plant pathogenesis, *A. euteiches *sequences were compared to the proteome of the fungal pathogen *Nectria haemetococca*.*Toxoplasma gondi *[20] and *Plasmodium falciparum *[21] proteomes were selected since it has been suggested recently that apicomplexan parasites and oomycetes share common infection strategies [22]. Finally, the proteome of *Arabidopsis thaliana *was also added to analysis.

Domain searches using InterProscan program (release 4.2) [23] were performed locally with HMM searches against Pfam protein database (16.0) [24]. 45% of sequences showed a known Pfam domain with an E-value ≤ 1e-5 (Table 1).

To classify the sequences according to the Gene Ontology classification scheme [25], Pfam domains with InterPro accession number were linked to GO molecular function, biological process and cellular component terms using the interpro2go file [26]. Finally, 42% of the sequences were assigned to a GO molecular function term, 43% to a biological process and 24% to a cellular component (Table 2).

**Table 2:** Classification of unigenes based on gene ontology (GO) mappings. Mappings of the InterPro domains to terms in the GO hierarchy were used to assign GO terms to the unigenes. Sequences for which a protein domain was predicted with an E value < 1e-5 were selected for this analysis.

**GO term**	***%***
**Biological process**	**43.07**
metabolic process	19.12
cellular process	14.97
establishment of localization	3.6
localization	3.6
biological regulation	0.95
response to stimulus	0.49
developmental process	0.29
biological adhesion	0.04
immune system process	0.01
multicellular organismal process	0.01
**Molecular function**	**41.53**
catalytic activity	20.71
binding	16.71
transporter activity	2.34
structural molecule activity	0.56
motor activity	0.36
transcription regulator activity	0.18
enzyme regulator activity	0.15
antioxidant activity	0.14
molecular transducer activity1	0.01
**Cellular component (%)**	**23.79**
cell	8.66
cell part	8.66
organelle	2.78
macromolecular complex	2.22
organelle part	1.02
extracellular region	0.34
envelope	0.06
membrane-enclosed lumen	0.04

Gene Ontology SQL database format was downloaded from the GO web site [27] and added to AphanoDB. Links between GO terms and the Enzyme Nomenclature terms [28] were established using ec2go mapping [29], and KEGG metabolic pathway map links [30] were also added to the database.

Repeat sequences were detected using RepeatMasker [31] with default parameters in order to identify microsatellite markers and result outputs were added to the database.

To estimate differences in gene expression levels between the saprophytic and the pathogenic growth conditions, a statistical analysis was performed based on EST frequency in each library. We used the test described by Susko et al. [32] to calculate the p-value and false discovery rate control (FDR) for multiple test correction [33] (false positive rate is controlled at less than α = 0.05).

### Database implementation

AphanoDB is a MySQL database containing features, cleaned ESTs and deduced contigs. At the end of each step of the AphanoDB pipeline, information was stored in the related table and linked to the core tables containing EST and consensus sequences. The dynamic structure enables addition of new sequences of different oomycete species and associated features. The database is available through an Apache Web server running on Fedora core 6 Linux. The web interface is based on the PHP, JavaScript and HTML languages; it enables dynamic MySQL queries with a user-friendly graphical interface.

## Utility and discussion

### User interface

**Figure 2:**
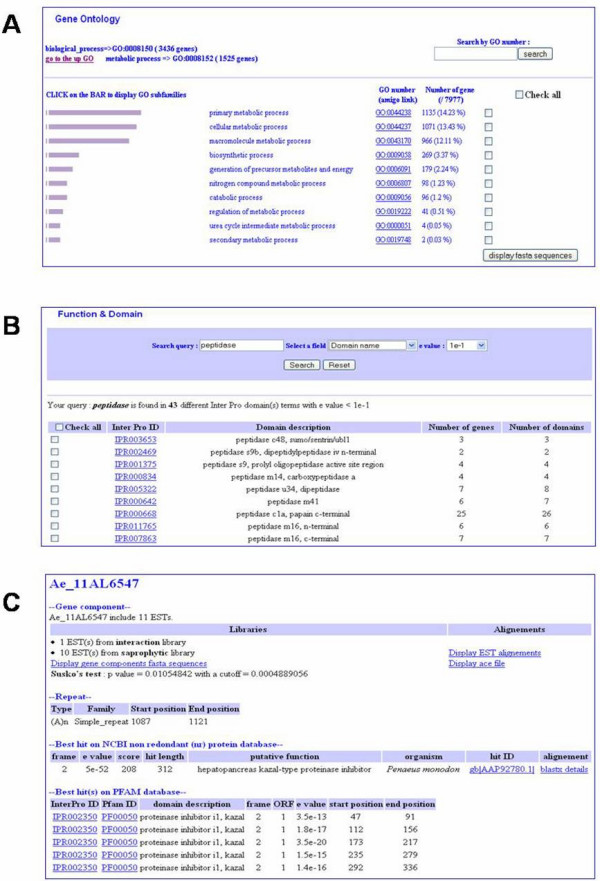
**Web interface**. A. Hierarchical browsing of Ontologies including distribution of the genes in the subcategories. B. Domain description query output with e value cut-off. First output clusters the genes by InterPro domain prediction. C. Part of gene report sheet.

AphanoDB provides a complete summary sheet containing all annotation results for each predicted unigene (Figure 2). The BLASTX results against the nr database, HMM search results against Pfam database and the Gene Ontology assignments are displayed on this summary sheet. BLASTX alignment details are also provided and will be updated yearly. The summary sheet also contains the sequence of the unigene, the link to the ESTs alignment, to the Ace file and another link to a multiFASTA file of the contig components.

Users can query the database for a given sequence by providing its accession number, EST name or gene ID. Searches on the Gene Ontology annotation are possible with graphical bars representing GO subfamilies (Figure 2A). For the GO catalytic activity subfamilies, EC links and KEGG metabolic pathway map links are provided. Function searches are allowed by queries by InterPro or Pfam domain names or accession numbers. BLASTX results stored in the database can be queried using organism name or putative function (Figure 2B–C). Queries on repeat sequence types and sizes are also available as well as on expression profile in a specific growth condition. For expression profiles, the output shows only significant results with a P-value lower than the Benjamini and Hochberg cut-off [33]. Users can submit their own sequences for BLAST searches against the *A. euteiches *sequences using BLASTN, TBLASTN and TBLASTX. The BLAST output page displays one summary sheet for each hit on the *A. euteiches *sequences.

### Utilities and extensions

AphanoDB provides molecular data about *A. euteiches *transcripts. The database contains 18,684 high quality sequences and allows direct applications for functional and comparative genomic approaches. AphanoDB is constructed in such a way that it can absorb a large number of additional sequences from other oomycete species, such as *S. parasitica*, for which large scale cDNA sequencing projects are under way.

## Conclusion

AphanoDB represents a major contribution to assist genomic studies on Oomycetes and other related organisms such as diatoms and brown algae. Addition of new sequences from other *Aphanomyces *species and other Saprolegniales is planned in the near future. AphanoDB will facilitate gene prediction and annotation for the future whole genome sequencing of Saprolegniales species. AphanoDB contains cleaned, assembled and annotated ESTs which will serve the oomycete research community. The database provides comprehensive tools for comparative approaches that might lead for example to leading to the identification of pathogenicity factors.

## Availability and requirements

AphanoDB is freely available to academic and non academic users at . A browser supporting frames must be used (Firefox, Netscape 2.0, Internet Explorer 3.0 or higher). cDNA clones can be obtained upon request at . All ESTs can be downloaded from AphanoDB. They have been also deposited in dbEST (accession numbers CU357053 to CU361296).

## List of abbreviations

EST: expressed sequence tag

GO: Gene Ontology

cDNA: complementary DNA

nr: non redondant

KEGG: Kyoto Encyclopedia of Genes and Genomes

HMM: Hidden Markov Model

## Authors' contributions

MAM designed the database and the web interface and wrote the pipeline programs. EG constructed the *Aphanomyces euteiches *cDNA libraries and coordinated the sequencing project. CM and HSC cooperated in structuring database and designed the pipeline draft. AC and PW managed the sequencing of the cDNA clones. BD led the AphanoDB project, helped to draft the manuscript and participated to the design of the database. All authors tested the database and revised the manuscript.

## References

[B1] Fry W, Goodwin S (1997). Re-emergence of potato and tomato late blight in the United States. Plant Dis.

[B2] Fry W, Smart C (1999). The return of *Phytophthora infestans*, a potato pathogen that just won't quit. Potato Res.

[B3] van West P (2006). *Saprolegnia parasitica*, an oomycete pathogen with a fishy appetite: new challenges for an old problem. Mycologist.

[B4] Jönsson-Belyazio U, Rosengren U (2006). Can *Phytophthora quercina* have a negative impact on mature pedunculate oaks under field conditions?. Ann For Sci.

[B5] Dick M (2001). In Straminipilous Fungi.

[B6] Kamoun S (2001). Nonhost resistance to *Phytophthora*: novel prospects for a classical problem. Curr Opin Plant Biol.

[B7] Tyler BM, Tripathy S, Zhang X, Dehal P, Jiang RH, Aerts A, Arredondo FD, Baxter L, Bensasson D, Beynon JL, Chapman J, Damasceno CM, Dorrance AE, Dou D, Dickerman AW, Dubchak IL, Garbelotto M, Gijzen M, Gordon SG, Govers F, Grunwald NJ, Huang W, Ivors KL, Jones RW, Kamoun S, Krampis K, Lamour KH, Lee MK, McDonald WH, Medina M, Meijer HJ, Nordberg EK, Maclean DJ, Ospina-Giraldo MD, Morris PF, Phuntumart V, Putnam NH, Rash S, Rose JK, Sakihama Y, Salamov AA, Savidor A, Scheuring CF, Smith BM, Sobral BW, Terry A, Torto-Alalibo TA, Win J, Xu Z, Zhang H, Grigoriev IV, Rokhsar DS, Boore JL (2006). *Phytophthora* genome sequences uncover evolutionary origins and mechanisms of pathogenesis. Science.

[B8] Govers F, Gijzen M (2006). *Phytophthora* genomics: the plant destroyers' genome decoded. Mol Plant Microbe Interact.

[B9] Gajendran K, Gonzales MD, Farmer A, Archuleta E, Win J, Waugh ME, Kamoun S (2006). *Phytophthora* functional genomics database (PFGD): functional genomics of *Phytophthora*-plant interactions. Nucleic Acids Res.

[B10] Torto-Alalibo TA, Tripathy S, Smith BM, Arredondo FD, Zhou L, Li H, Chibucos MC, Qutob D, Gijzen M, Mao C, Sobral BW, Waugh ME, Mitchell TK, Dean RA, Tyler BM (2007). Expressed sequence tags from *Phytophthora sojae* reveal genes specific to development and infection. Mol Plant Microbe Interact.

[B11] Randall TA, Dwyer RA, Huitema E, Beyer K, Cvitanich C, Kelkar H, Fong AM, Gates K, Roberts S, Yatzkan E, Gaffney T, Law M, Testa A, Torto-Alalibo T, Zhang M, Zheng L, Mueller E, Windass J, Binder A, Birch PR, Gisi U, Govers F, Gow NA, Mauch F, van West P, Waugh ME, Yu J, Boller T, Kamoun S, Lam ST, Judelson HS (2005). Large-scale gene discovery in the oomycete *Phytophthora infestans* reveals likely components of phytopathogenicity shared with true fungi. Mol Plant Microbe Interact.

[B12] Torto-Alalibo T, Tian M, Gajendran K, Waugh ME, van West P, Kamoun S (2005). Expressed sequence tags from the oomycete fish pathogen *Saprolegnia parasitica* reveal putative virulence factors. BMC Microbiol.

[B13] Gaulin E, Jacquet C, Bottin A, Dumas B (2007). Root rot disease of legumes caused by *Aphanomyces euteiches*. Molecular Plant Pathol.

[B14] Malvick D, Grau C (2001). Characteristics and frequency of *Aphanomyces euteiches* races 1 and 2 associated with alfalfa in the Midwestern United States. Plant dis.

[B15] Petersen A (1996). Use of polyclonal antibodies to detect oospores of *Aphanomyces*. Mycol Res.

[B16] Ewing B, Green P (1998). Base-calling of automated sequencer traces using Phred. II. Error probabilities. Genome Res.

[B17] Huang X, Madan A (1999). CAP3: a DNA sequence assembly program. Genome Res.

[B18] Altschul SF, Madden TL, Schaffer AA, Zhang J, Zhang Z, Miller W, Lipman DJ (1997). Gapped BLAST and PSI-BLAST: a new generation of protein database search programs. Nucleic Acids Res.

[B19] Armbrust EV, Berges JA, Bowler C, Green BR, Martinez D, Putnam NH, Zhou S, Allen AE, Apt KE, Bechner M, Brzezinski MA, Chaal BK, Chiovitti A, Davis AK, Demarest MS, Detter JC, Glavina T, Goodstein D, Hadi MZ, Hellsten U, Hildebrand M, Jenkins BD, Jurka J, Kapitonov VV, Kroger N, Lau WW, Lane TW, Larimer FW, Lippmeier JC, Lucas S, Medina M, Montsant A, Obornik M, Parker MS, Palenik B, Pazour GJ, Richardson PM, Rynearson TA, Saito MA, Schwartz DC, Thamatrakoln K, Valentin K, Vardi A, Wilkerson FP, Rokhsar DS (2004). The genome of the diatom *Thalassiosira pseudonana*: ecology, evolution, and metabolism. Science.

[B20] Kissinger JC, Gajria B, Li L, Paulsen IT, Roos DS (2003). ToxoDB: accessing the Toxoplasma gondii genome. Nucleic Acids Res.

[B21] Bahl A, Brunk B, Crabtree J, Fraunholz MJ, Gajria B, Grant GR, Ginsburg H, Gupta D, Kissinger JC, Labo P, Li L, Mailman MD, Milgram AJ, Pearson DS, Roos DS, Schug J, Stoeckert CJ, Whetzel P (2003). PlasmoDB: the Plasmodium genome resource. A database integrating experimental and computational data. Nucleic Acids Res.

[B22] Bhattacharjee S, Hiller NL, Liolios K, Win J, Kanneganti TD, Young C, Kamoun S, Haldar K (2006). The malarial host-targeting signal is conserved in the Irish potato famine pathogen. PLoS Pathog.

[B23] Zdobnov EM, Apweiler R (2001). InterProScan - an integration platform for the signature-recognition methods in InterPro. Bioinformatics.

[B24] Bateman A, Coin L, Durbin R, Finn RD, Hollich V, Griffiths-Jones S, Khanna A, Marshall M, Moxon S, Sonnhammer EL, Studholme DJ, Yeats C, Eddy SR (2004). The Pfam protein families database. Nucleic Acids Res.

[B25] Harris MA, Clark J, Ireland A, Lomax J, Ashburner M, Foulger R, Eilbeck K, Lewis S, Marshall B, Mungall C, Richter J, Rubin GM, Blake JA, Bult C, Dolan M, Drabkin H, Eppig JT, Hill DP, Ni L, Ringwald M, Balakrishnan R, Cherry JM, Christie KR, Costanzo MC, Dwight SS, Engel S, Fisk DG, Hirschman JE, Hong EL, Nash RS, Sethuraman A, Theesfeld CL, Botstein D, Dolinski K, Feierbach B, Berardini T, Mundodi S, Rhee SY, Apweiler R, Barrell D, Camon E, Dimmer E, Lee V, Chisholm R, Gaudet P, Kibbe W, Kishore R, Schwarz EM, Sternberg P, Gwinn M, Hannick L, Wortman J, Berriman M, Wood V, de la Cruz N, Tonellato P, Jaiswal P, Seigfried T, White R (2004). The Gene Ontology (GO) database and informatics resource. Nucleic Acids Res.

[B26] interpro2go file. http://www.geneontology.org/external2go/interpro2go.

[B27] Gene Ontology SQL database. http://www.geneontology.org/GO.downloads.shtml.

[B28] (1999). Nomenclature committee of the international union of biochemistry and molecular biology (NC-IUBMB), Enzyme Supplement 5 (1999). Eur J Biochem.

[B29] ec2go file. http://www.geneontology.org/external2go/ec2go.

[B30] Kanehisa M (2002). The KEGG database. Novartis Found Symp.

[B31] Bedell JA, Korf I, Gish W (2000). MaskerAid: a performance enhancement to RepeatMasker. Bioinformatics.

[B32] Susko E, Roger AJ (2004). Estimating and comparing the rates of gene discovery and expressed sequence tag (EST) frequencies in EST surveys. Bioinformatics.

[B33] Benjamini Y, Hochberg Y (1995). Controlling the false discovery rate: a practical and powerful approach to multiple testing. J R Statist Soc.

